# Micro-nanobubble oxygenation irrigation ameliorates saline-alkali soil properties, cotton physiology, and yield under different salt stress levels

**DOI:** 10.3389/fpls.2026.1727907

**Published:** 2026-02-06

**Authors:** Yongxia Yang, Qingyong Bian, Yaozu Feng, Zhiguo Wang, Yanbo Fu, Yanhong Wei, Jinquan Zhu

**Affiliations:** 1 Institute of Microbiology, Xinjiang Academy of Agricultural Sciences, Urumqi, China; 2 College of Resources and Environment, Xinjiang Agricultural University, Urumqi, China; 3 Institute of Agricultural Resources and Environment, Xinjiang Academy of Agricultural Sciences, Urumqi, China; 4 Baicheng Base, Xinjiang Academy of Agricultural Sciences, National Soil Quality Aksu Field Scientific Observation and Research Station, Ministry of Agriculture and Rural Affairs, Aksu, Xinjiang, China

**Keywords:** antioxidant enzymes, cotton growth, microbial community, micro-nanobubble oxygenation irrigation, salt tolerance, soil salinity, sustainable agriculture

## Abstract

**Background:**

Addressing soil saline-alkalization is crucial for sustaining cotton production in the arid regions of Xinjiang. This study investigates the efficacy of Micro-Nanobubble oxygenated irrigation (MNBs) compared with conventional flooding (CF) in ameliorating saline-alkali soil and enhancing cotton growth.

**Methods:**

A field microplot experiment was conducted across four soil salinity levels (0, 3%, 6%, and 9%, with sulfate as the dominant salt).

**Results:**

The results demonstrated that MNBs effectively reduced topsoil (0~20 cm) salinity and mitigated its associated alkalinity stress by facilitating salt leaching into deeper soil layers (20~60 cm). This irrigation method also significantly improved soil enzyme activities and altered ionic dynamics toward a more favorable balance. Moreover, MNBs enhanced soil bacterial diversity, enriched beneficial phyla such as Proteobacteria and Actinobacteria, and modulated fungal genera including Alternaria and Fusarium, suggesting an improved rhizospheric microbiome. In terms of cotton physiology, Micro-nanobubble oxygenation irrigation significantly enhanced the activities of superoxide dismutase (SOD) and peroxidase (POD) in cotton leaves by 15.84% to 40.69% and 10.11% to 33.63%, respectively, while reducing malondialdehyde (MDA) content by 28.22% to 42.11%, thereby alleviating saline-alkali stress-induced oxidative damage. Additionally, MNBs promoted root growth by 0.96% to 29.90%, increased the leaf area index by 18.68% to 25.50%, and enhanced dry matter accumulation by 6.82% to 33.29%. Ultimately, these improvements led to a higher seed cotton yield. Compared with conventional flooding (CF), the MNBs treatment increased seed cotton yield by 33.78%, 35.93%, 47.11%, and 52.31% across the four salinity levels, respectively.

**Conclusion:**

In conclusion, micro-nanobubble oxygenation irrigation represents an effective strategy for rehabilitating saline-alkali soils and promoting sustainable agricultural development in arid areas.

## 1 Introduction

Soil salinization has emerged as a major challenge to agricultural sustainability in arid and semi-arid regions worldwide (Zhou et al., 2023). In China, over 90% of cotton is produced in Xinjiang, and the stability of this core production area is critical to ensuring the security of the national textile raw material supply. Although cotton is considered a moderately salt-tolerant pioneer crop, its growth and productivity remain significantly constrained by the adverse characteristics of saline-alkali soils. Specifically, excess sodium ions disperse soil colloids and destroy aggregate structure, leading to severe compaction, reduced porosity, and markedly impaired gas permeability (Li et al., 2025; Sikder and Khan, 2024). The associated high pH environment exacerbates nutrient fixation and imbalance, which, coupled with an oxygen-deficient rhizosphere, creates a compounded stress complex that fundamentally undermines soil function (Russo et al., 2020). Current amelioration strategies for soil salinization predominantly rely on water leaching for salt removal (Baloch et al., 2025). However, the widely adopted mulch-drip irrigation in this region, while saving water and increasing yield, tends to cause unidirectional water movement that leads to salt accumulation in the plow layer, thereby inducing secondary salinization (Zhang et al., 2022). Therefore, there is an urgent need to develop advanced irrigation technologies capable of effectively improving saline-alkali soils and overcoming the aforementioned constraints—both in Xinjiang and in similar ecoregions globally.

In recent years, micro-nanobubble irrigation technology has emerged as a promising innovation in agricultural water management, drawing increasing attention for its unique advantages in regulating root zone microenvironments. By virtue of high-speed rotational shear technology, gases like air, oxygen, or ozone are fragmented into ultrafine bubbles with diameters <50 μm (Li et al., 2014). These micro-nanobubbles exhibit distinct characteristics: a large specific surface area and extremely low rise velocity, which extend their residence time in irrigation water and promote efficient gas-liquid mass transfer. This not only effectively elevates dissolved oxygen levels in the root zone to alleviate hypoxia stress but also provides a theoretical basis for mitigating plant salt stress through improved root respiration and physiological metabolism (Wang et al., 2024a, Wang et al., 2023). As a green and efficient irrigation strategy, it holds significant potential for application in saline-alkali land improvement and high-yield crop cultivation, offering new technical support for sustainable agricultural development.

In cotton plants, the detrimental effects of salt stress manifest as a typical cascade from subcellular structures to overall agronomic traits. This process initiates at the cellular and molecular levels, where excessive Na^+^ in the rhizosphere intrudes into the cytoplasm, disrupts K^+^ homeostasis, and impairs mitochondrial and chloroplast functions. This subsequently triggers a burst accumulation of reactive oxygen species (ROS), leading to oxidative damage (Ratcliffe and Shachar-Hill, 2010). In response, the plant activates defense mechanisms, including the SOS ion efflux pathway and the CBL-CIPK calcium signaling network, to reestablish homeostasis and induce adaptive gene expression (Ratcliffe et al., 2010). Such fundamental cellular disturbances directly result in dysfunction at the physiological and biochemical levels, characterized by dynamic changes in the activities of antioxidant enzymes such as SOD, POD, and CAT (Dong et al., 2002), accumulation of osmoregulatory substances like proline (Büra and Leblebici, 2025), and a significant decline in photosynthetic rate due to stomatal limitation and inhibition of chlorophyll synthesis (Rathod and Anand, 2015). Ultimately, all these micro-level dysregulations are integrated and amplified at the whole-plant level. Root development and biomass accumulation are severely inhibited, shoot growth is stunted, and during the reproductive stage, increased bud and boll shedding, reduced boll number and weight are observed, collectively leading to substantial losses in seed cotton yield and fiber quality.

Although research on the intrinsic salt tolerance mechanisms of cotton has been continuously advancing, studies on how to proactively intervene and alleviate the aforementioned multi-level stress damages through external agronomic measures remain insufficient. In particular, the efficacy of Micro-Nanobubble Oxygenation Irrigation (MNBs)—a novel physical technology designed to improve the root-zone oxygen environment—in ameliorating saline-alkali cotton field systems, especially under typical field conditions as represented by Xinjiang, is still unclear. There is a lack of systematic empirical research investigating whether and how MNBs can positively influence the entire physiological cascade of cotton, from ion homeostasis and antioxidant defense to photosynthetic production and ultimately yield, by regulating soil physicochemical properties and microbial activity.

Therefore, this study aims to systematically evaluate the comprehensive effects of Micro-Nanobubble Oxygenation Irrigation on the properties of saline-alkali soils and cotton growth under different salinity levels through a rigorous field micro-plot experiment. The specific objectives are as follows:(1) To analyze the impact of Micro-Nanobubble Oxygenation Irrigation on the soil environment.(2) To investigate the effects of Micro-Nanobubble Oxygenation Irrigation on leaf enzyme activities and growth indicators of cotton grown in salinized soil.(3) To clarify the influence of Micro-Nanobubble Oxygenation Irrigation on cotton yield. It is expected that this study will provide new insights for improving and managing salinized soil environments and promoting cotton production.

## 2 Materials and methods

### 2.1 Study area overview

The experiment was conducted in 2024 at the Akesu Field Scientific Observation Station for Soil Quality under the Ministry of Agriculture and Rural Affairs (41°48′N, 81°54′55″E), at an elevation of 1200 m. The climate is temperate continental arid, with an annual mean temperature of 7.6°C, maximum temperature of 38.3°C, and minimum temperature of -28°C. The frost-free period ranges from 133 to 163 days, annual sunshine duration averages 2789.7 hours, and annual precipitation averages 171.13 mm. The physicochemical properties of the rhizosphere soil are summarized in (**Table 1**).

**Table 1:** Soil physicochemical properties of the rhizosphere.

Soil types	pH	Water-soluble salt (g·kg^-1^)	Hydrolyzable nitrogen (mg·kg^-1^)	Available phosphorus (mg·kg^-1^)	Quick-acting potassium (mg·kg^-1^)	Organic matter (mg·kg^-1^)	Soil bulk density (g·cm^3^)	Soil porosity (%)
Clay	7.91	0.07	81.4	14.7	171	13.66	1.43	37

### 2.2 Experimental design

**Figure 1:**
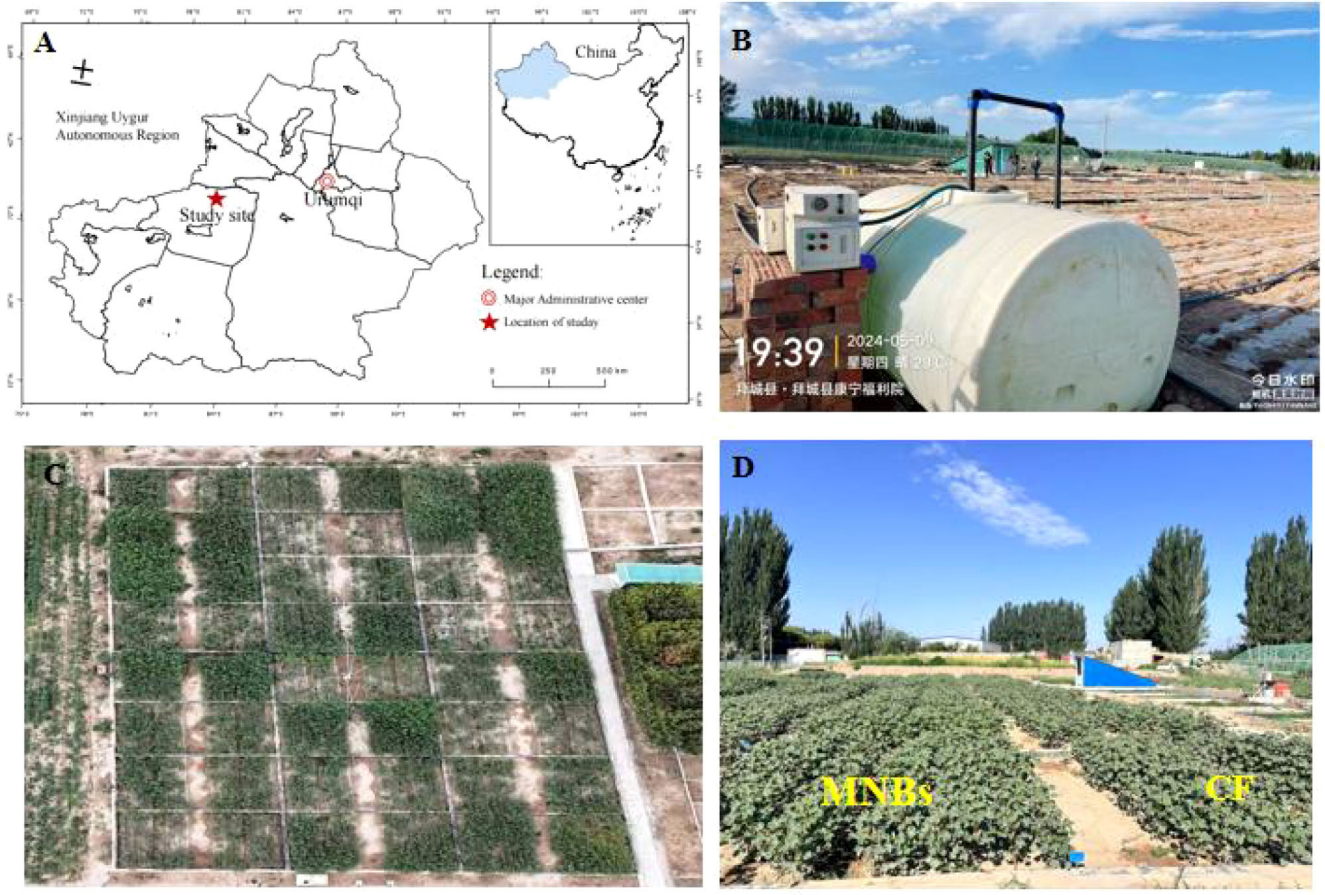
Experimental layout. **(A)** Location and layout of the study area; **(B)** Irrigation equipment; **(C, D)** Experimental treatments. The experimental site is located in southern Xinjiang, China. The experiment primarily employed saline-alkali soil cultivation, with irrigation equipment consisting of three main components: Micro-Nanobubble Oxygenation Irrigation devices, oxygen supply systems, and water sources. The experimental design comprised two irrigation methods and four soil salinity stress levels: Micro-Nanobubble Oxygenation Irrigation treatments MNBs-S_0_, MNBs-S_0.3_, MNBs-S_0.6_, MNBs-S_0.9_; and conventional water irrigation treatments CF-S_0_, CF-S_0.3_, CF-S_0.6_, CF-S_0.9_.

Test soils were collected from the 0~20 cm soil layer at the Akesu Field Scientific Observation Station for Soil Quality under the Ministry of Agriculture and Rural Affairs. After collection, debris was removed, and the soil underwent natural air-drying, crushing, and sieving through a 1 cm mesh. It was then mixed according to the experimental soil salinity levels. The test cotton variety was “J026-5” (provided by Xinjiang Academy of Agricultural Sciences). Seeds were manually spot-sown on May 7, 2024, with sampling completed on October 15. The “dry sowing, wet emergence” method was employed under a “1-film, 3-pipe, 6-row” planting pattern: wide rows spaced 66 cm apart, narrow rows 10 cm apart, and plants spaced 11 cm apart. The experiment utilized saline micro-plots, each measuring 10 m (length) × 5 m (width) × 1 m (height). Four salinity gradients were established: no salt (S_0_), 3 g·kg-¹ (S_0.3_), 6 g·kg ^-1^ (S_0.6_), and 9 g·kg^-1^ (S_0.9_). Two irrigation methods were applied: Micro-Nanobubble Oxygenation Irrigation (MNBs) and non-oxygenated irrigation (CF)). A completely randomized block design was employed, resulting in eight treatments: MNBs-S_0_, MNBs-S_0.3_, MNBs-S_0.6_, MNBs-S_0.9_, CF-S_0_, CF-S_0.3_, CF-S_0.6_, CF-S_0.9_), each with three replicates. Surface water from conventional irrigation systems, characterized by dissolved oxygen (DO) levels of 8–9 mg·L^-^¹, served as the source for irrigation. To elevate the oxygen content, a micro-nanobubble generator (B&W type, Benzhou (Beijing) New Technology Promotion Co., Ltd., China) was employed, operating at an inlet flow rate of 1.5 L·min^-^¹ and a pressure of 0.015 MPa. Further augmentation of dissolved oxygen was achieved by integrating an oxygen supply unit (YU300 model, Jiangsu Yuyue Medical Equipment Co., Ltd., China) delivering oxygen at a flow rate of 2 L·min^-^¹. The system was set to maximum oxygen supply (90% concentration) until DO levels stabilized. A portable dissolved oxygen meter (Seven2Go™, Mettler Toledo International Trading Co., Ltd., Shanghai, China; accuracy ±0.1 mg·L^-^¹) was used to monitor the DO concentration, which reached a stable value of 30 mg·L^-^¹. The oxygen-enriched irrigation water was subsequently delivered directly to the root zone via a drip irrigation system (**Figure 1B**). Both irrigation volume and fertilization management throughout the growth cycle adhered to local conventional practices.

### 2.3 Measurement and application methods

#### 2.3.1 Cotton growth, physiological, and yield parameters

Root length: At maturity, three cotton plants per treatment were randomly selected for root characterization. Roots within a horizontal distance of 10 cm from the main stem and a vertical depth of 60 cm were carefully excavated. After cleaning and gently drying, the samples were scanned using an EPSON Expression 10000XL scanner. Root phenotypic traits, including root length, were analyzed with WinRhizo REG 2009 software (Canada).

Leaf area index (LAI) and dry matter mass (DM): Three representative plants from each plot were chosen for LAI measurement. All leaves were detached, arranged on a white plate, and photographed in JPG format. The images were processed with image analysis software to determine LAI. For DM assessment, roots, stems, leaves, and reproductive organs were separated, heated at 105°C for 30 min, and then dried at 75°C until constant weight was achieved. The dried samples were weighed after cooling (Wang et al., 2024).

Leaf enzyme activities and malondialdehyde content: Fresh, fully expanded functional leaves were collected at key growth stages of cotton, snap-frozen in liquid nitrogen, and stored at –80°C until analysis. Frozen leaf samples were weighed and homogenized in ice-cold phosphate buffer. The homogenate was centrifuged to obtain the crude enzyme extract. Superoxide dismutase (SOD) activity was determined using the nitroblue tetrazolium (NBT) method (Qu et al., 2024). Peroxidase (POD) activity was assayed by the guaiacol method (Gentile et al., 2023). Malondialdehyde (MDA) content was measured using the thiobarbituric acid (TBA) colorimetric method (Min et al., 2023).

Yield: Cotton yield was evaluated at full maturity using whole-plot harvesting. The total number of bolls exceeding 2 cm in diameter was recorded for each treatment. A subsample of 100 bolls was collected from each plot and weighed to determine average boll mass. Seed cotton yield was calculated as the product of total boll number and average boll mass (Escobar et al., 2021).

#### 2.3.2 Soil salinity

Soil samples were collected at the seedling, bud, flowering, and boll-setting, and maturity stages of cotton using a five-point sampling method. Using a soil auger, random samples were taken from the 0~20 cm soil layer under the plant root zone, then mixed thoroughly to form a composite sample. The samples were air-dried, ground, and passed through a 2 mm sieve. A 20 g aliquot of the processed soil was mixed with deionized water at a 1:5 soil-to-water ratio, shaken for 0.5 h on a mechanical shaker, and soil soluble salt content was determined gravimetrically (Jin et al., 2024; Kargas et al., 2018; Hu et al., 2020).

#### 2.3.3 Soil enzyme activity and microbial sequencing

Given that microbial diversity has been shown to increase and community structure to become more homogeneous as crops mature (Urlić et al., 2023), rhizosphere soil samples were collected during the cotton maturation stage. From each treatment, three plants were randomly selected. Rectangular soil blocks (20 cm × 20 cm × 40 cm) were excavated vertically following the root system. After gently removing loose soil by shaking, the adhered rhizosphere soil was carefully brushed from the roots into sterile bags and immediately transported to the laboratory. The soil was divided into two aliquots: one was refrigerated for enzyme activity assays, and the other was stored at –80°C for microbial sequencing.

##### 2.3.3.1 Measurement of soil enzyme activity

Soil alkaline phosphatase (ALP) activity was determined using the Tabatabai method (Alef and Nannipieri, 1995), based on the release of p-nitrophenol from nitrophenyl phosphate (PNPP) and measured at 410 nm. Soil sucrase (SU) activity was assayed by quantifying reducing sugars using the 3,5-dinitrosalicylic acid (DNS) method, with absorbance read at 540 nm. Catalase activity was evaluated by measuring the decomposition of H_2_O_2_ via titration of residual H_2_O_2_ with potassium permanganate. Urease (URE) activity was determined colorimetrically with Nessler’s reagent, measuring ammonia release at 630 nm (Chen et al., 2018).

##### 2.3.3.2 PCR amplification and highthroughput sequencing

Fresh rhizosphere soil samples (0.5 g) were subjected to DNA extraction using the E.Z.N.A.® Soil DNA Kit (Omega Bio-tek, Norcross, GA, U.S.). DNA concentration and purity were assessed with a NanoDrop 2000 spectrophotometer (Bio-Rad Laboratories Inc., USA).

The V3–V4 region of the bacterial 16S rRNA gene was amplified with primers 338F (5'-ACTCCTACGGGAGGCAGCA-3') and 806R (5'-GGACTACHVGGGTWTCTAAT-3'), and the fungal ITS1 region with primers ITS5F (5'-GGAAGTAAAAGTCGTAACAAGG-3') and ITS1R (5'-GCTGCGTTCTTCATCGATGC-3'). The 25 µL PCR mixture consisted of 5 µL 5× reaction buffer, 4 µL 5× GC buffer, 2 µL of 2.5 mM dNTPs, 1 µL each of forward and reverse primers (10 mM), 2 µL DNA template, 0.25 µL Q5 Hi-Fi DNA polymerase, and ddH_2_O to volume. Thermal cycling conditions were: 95°C for 2 min; 25–30 cycles of 98°C for 15 s, 55°C for 30 s, and 72°C for 30 s; followed by 72°C for 5 min.

Amplicons were sequenced on the Illumina MiSeq PE300 platform (Magoč and Salzberg, 2011). Raw reads were quality-controlled using fastp (v0.20.0) and assembled with FLASH (v1.2.7) (Edgar, 2013). High-quality tags were obtained after filtering with Trimmomatic (v0.33). UPARSE (v7.1) was used to cluster non-redundant sequences into operational taxonomic units (OTUs) at 97% similarity (Stackebrandt and Goebel, 1994), during which chimeras and singletons were removed. Clean reads were mapped to representative OTU sequences at ≥97% identity. Bacterial and fungal datasets were analyzed separately for diversity and community composition.

### 2.4 Statistical analysis

All data were preprocessed using Excel 2016 (Microsoft, Redmond, USA), SPSS Statistics 27 (IBM, Armonk, USA), and Origin 2021 (OriginLab, Northampton, USA), while the experimental area was mapped with ArcGIS. Bar graphs illustrated the effects of treatments on soil salinity. Variations in soil enzyme activity and microbial diversity indices across treatments were displayed using box plots, and microbial community composition was visualized through stacked bar plots.

## 3 Results

### 3.1 Soil salinity

**Figure 2:**
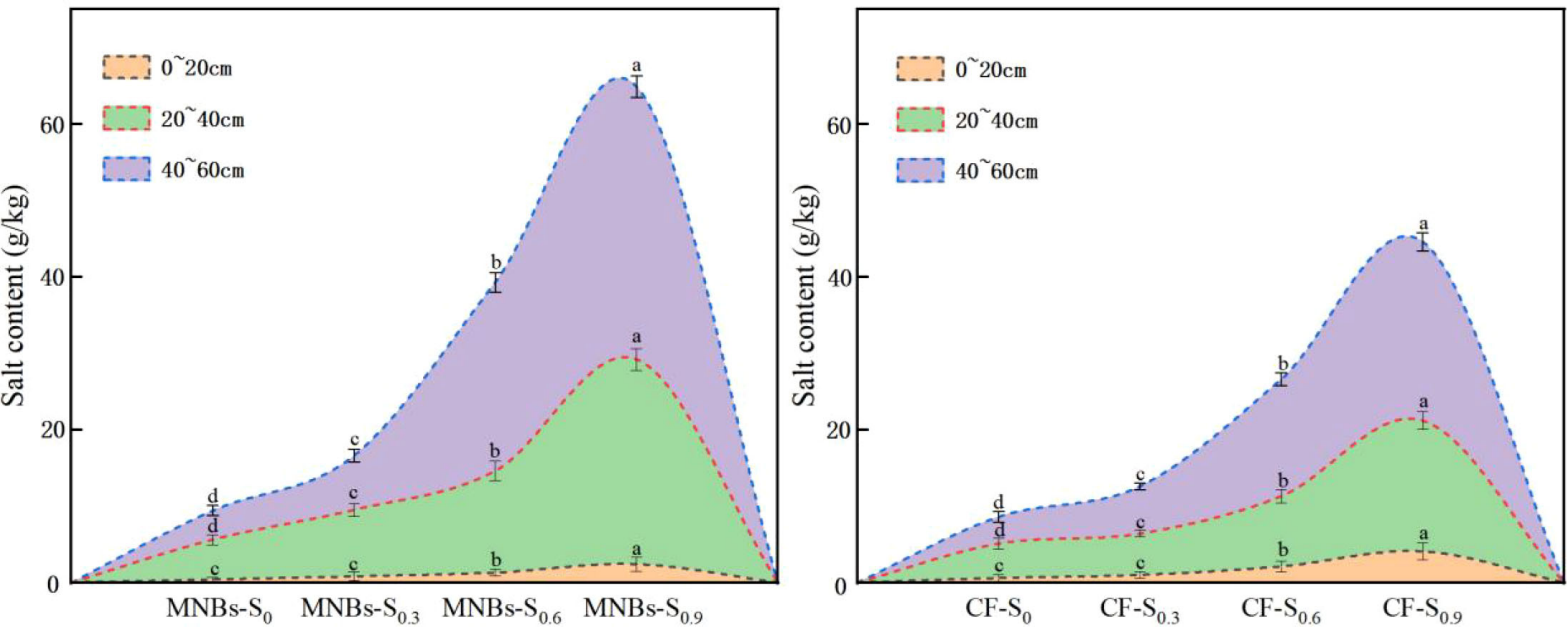
Effects of different treatments on soil salinity in cotton. Different lowercase letters (a–c) indicate significant differences (*P* < 0.05) in the mean soil salinity levels among treatments at different growth stages of cotton. Identical lowercase letters indicate no significant differences (*P* < 0.05) in the mean soil salinity levels among treatments at different growth stages of cotton.

To investigate the effects of different irrigation treatments on soil salinity in saline cotton fields (**Figure 2**), soil salinity in the top layer (0~20 cm) exhibited a gradual decrease across all eight treatments as the cotton growth period advanced, while an increasing trend was observed in the deeper soil layer (40~60 cm). Among these, the Micro-Nanobubble Oxygenation Irrigation (MNBs) treatment resulted in a more pronounced reduction in topsoil salinity. Furthermore, this treatment promoted the accumulation of salts in the deeper soil profile.

Compared to the non-oxygenated control group, the four MNBs treatments (MNBs-S_0,_ MNBs-S_0.3_, MNBs-S_0.6_, and MNBs-S_0.9_) significantly reduced salinity in the surface soil (0~20 cm) by 20.00%, 33.33%, 38.10%, and 41.46%, respectively (*P* < 0.05). Concurrently, salinity in the subsurface layer (40~60 cm) increased by 8.57%, 14.52%, 52.56%, and 61.44% (*P* < 0.05). These results demonstrate that Micro-Nanobubble Oxygenation Irrigation facilitates the downward leaching of salts, thereby effectively mitigating surface soil salinization.

### 3.2 pH

**Figure 3:**
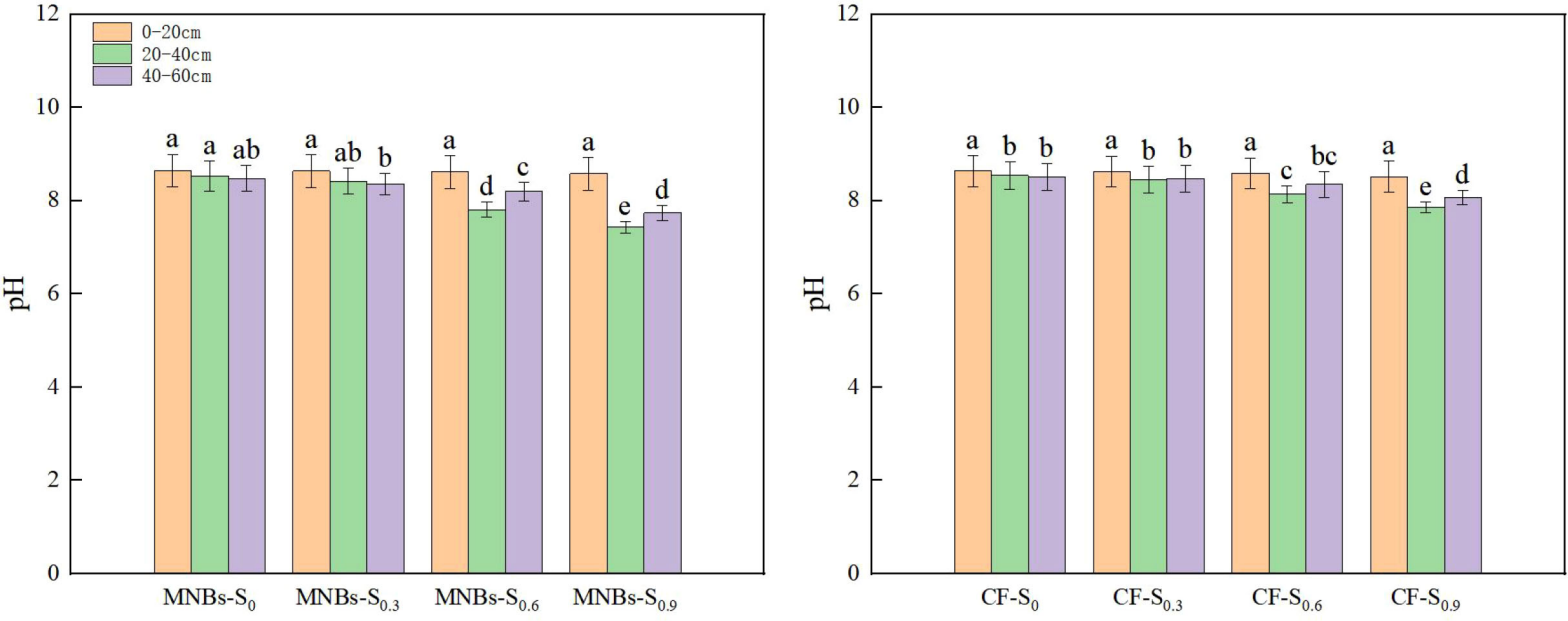
Effects of different treatments on soil salinity in cotton. Different lowercase letters (a–c) indicate significant differences (*P* < 0.05) in the mean soil salinity levels among treatments at different growth stages of cotton. Identical lowercase letters indicate no significant differences (*P* < 0.05) in the mean soil salinity levels among treatments at different growth stages of cotton.

To investigate the effects of different treatments on soil pH in saline-alkali cotton fields (**Figure 3**), with increasing soil depth, the pH in the surface soil remained relatively stable under the eight treatments, while the pH in the deeper soil layer (40~60 cm) gradually decreased. The reduction in pH was more pronounced in the deep soil under micro-nanobubble oxygenation irrigation treatments. Moreover, micro-nanobubble oxygenation irrigation promoted the downward migration of alkaline substances in the soil. Compared with the non-oxygenated treatments, the four micro-nanobubble oxygenation irrigation treatments (MNBs-S_0_, MNBs-S_0_._3_, MNBs-S_0_._6_, and MNBs-S_0_._9_) increased the surface soil (0~20 cm) pH by 0.12 %, 0.12 %, 0.35 %, and 0.70 %, respectively (*p* < 0.05), while decreasing the deep soil (40~60 cm) pH by 0.35 %, 1.42 %, 1.80 %, and 4.09 %, respectively (*p* < 0.05). These results indicate that micro-nanobubble oxygenation irrigation facilitates the downward movement of alkaline substances, thereby regulating soil pH and improving the environment of saline-alkali soil.

### 3.3 Soil salt ions

To investigate the effects of different treatments on soil ion content in saline cotton fields (**Tables 2**–**4**), the results demonstrated that salt concentration, irrigation method, and their interaction had highly significant effects (*P* < 0.05) on the concentrations of Ca²^+^, Mg²^+^, K^+^, Na^+^, HCO_3_^-^, Cl^-^, and SO_4_²^-^ in the 0~20 cm soil layer. In the 20~40 cm layer, both main factors and their interaction also significantly influenced the levels of Mg²^+^, K^+^, Na^+^, HCO_3_^-^, Cl^-^, and SO_4_²^-^ (*P* < 0.05). A significant interaction effect between salt concentration and irrigation method was similarly observed for Ca²^+^ in this layer (*P* < 0.05). In the deepest soil layer (40~60 cm), salt concentration, irrigation method, and their interaction significantly affected the concentrations of Na^+^, HCO_3_^-^, Cl^-^, and SO_4_²^-^ (*P* < 0.05).

**Table 2:** The 0~20 cm soil layer.

Treatment	HCO_3-_ ( mg·kg^-1^)	Cl^-^ ( mg·kg^-1^)	SO_4_^2-^ (mg·kg^-1^)	Ca^2+^ (mg·kg^-1^)	Mg^2+^ (mg·kg^-1^)	K^+^ (mg·kg^-1^)	Na^+^ (mg·kg^-1^)
MNBs-S_0_	0.11 ± 0.01f	0.21 ± 0.01f	0.42 ± 0.03f	0.30 ± 0.02c	0.08 ± 0.01e	0.05 ± 0.01d	0.01 ± 0.01f
CF-S_0_	0.14 ± 0.01e	0.31 ± 0.01e	0.56 ± 0.04e	0.33 ± 0.20c	0.17 ± 0.01c	0.06 ± 0.01c	0.05 ± 0.01d
MNBs-S_0.3_	0.21 ± 0.02d	0.32 ± 0.01e	0.63 ± 0.05d	0.34 ± 0.20c	0.09 ± 0.01d	0.06 ± 0.01c	0.02 ± 0.01e
CF-S_0.3_	0.23 ± 0.02d	0.42 ± 0.02d	0.81 ± 0.07c	0.39 ± 0.03b	0.10 ± 0.01d	0.06 ± 0.01c	0.06 ± 0.01c
MNBs-S_0.6_	0.24 ± 0.02c	0.43 ± 0.02d	0.73 ± 0.06c	0.40 ± 0.30b	0.18 ± 0.01c	0.06 ± 0.01c	0.03 ± 0.01e
CF-S_0.6_	0.27 ± 0.02b	0.75 ± 0.04b	1.04 ± 0.09b	0.52 ± 0.04a	0.17 ± 0.01c	0.07 ± 0.01b	0.08 ± 0.01b
MNBs-S_0.9_	0.25 ± 0.02c	0.54 ± 0.03c	0.82 ± 0.07c	0.41 ± 0.04b	0.20 ± 0.02b	0.06 ± 0.01c	0.04 ± 0.01d
CF-S_0.9_	0.3 ± 0.03a	1.01 ± 0.06a	1.33 ± 0.10a	0.55 ± 0.05a	0.28 ± 0.03a	0.08 ± 0.01a	0.10 ± 0.01a
Soil Salinity	***	***	***	**	***	***	***
Irrigation Method	***	***	***	**	***	***	***
Soil Salinity ×Irrigation Method	***	***	***	*	***	***	***

* indicates *P* < 0.05 significant level; ** indicates the extremely significant level of *P* < 0.01, the same below. Data are means ± SD (n = 3).

**Table 3:** The 20~40 cm soil layer.

Treatment	HCO_3-_ ( mg·kg^-1^)	Cl^-^ ( mg·kg^-1^)	SO_4_^2-^ (mg·kg^-1^)	Ca^2+^ (mg·kg^-1^)	Mg^2+^ (mg·kg^-1^)	K^+^ (mg·kg^-1^)	Na^+^ (mg·kg^-1^)
MNBs-S_0_	0.38 ± 0.02e	0.88 ± 0.06h	1.98 ± 0.16e	0.36 ± 0.02f	0.14 ± 0.01e	0.12 ± 0.01f	0.67 ± 0.04h
CF-S_0_	0.40 ± 0.02d	1.34 ± 0.10f	2.31 ± 0.2d	0.37 ± 0.02f	0.16 ± 0.01d	0.13 ± 0.01f	1.01 ± 0.08f
MNBs-S_0.3_	0.39 ± 0.03e	1.01 ± 0.09g	2.11 ± 0.19d	0.78 ± 0.05e	0.18 ± 0.01d	0.14 ± 0.01e	0.82 ± 0.06g
CF-S_0.3_	0.41 ± 0.03d	1.56 ± 0.11e	2.54 ± 0.23c	0.81 ± 0.06d	0.20 ± 0.02c	0.15 ± 0.01e	1.46 ± 0.10d
MNBs-S_0.6_	0.42 ± 0.03d	1.69 ± 0.13d	3.23 ± 0.28b	0.80 ± 0.06d	0.22 ± 0.02c	0.17 ± 0.01d	1.23 ± 0.09e
CF-S_0.6_	0.46 ± 0.04b	2.23 ± 0.17b	2.72 ± 0.24c	0.83 ± 0.06c	0.23 ± 0.02b	0.19 ± 0.01c	2.61 ± 0.21b
MNBs-S_0.9_	0.45 ± 0.04c	2.01 ± 0.16c	4.10 ± 0.34a	0.91 ± 0.07b	0.26 ± 0.03a	0.21 ± 0.02b	2.31 ± 0.19c
CF-S_0.9_	0.53 ± 0.05a	3.16 ± 0.24a	3.14 ± 0.26b	0.98 ± 0.08a	0.27 ± 0.03a	0.22 ± 0.02a	3.11 ± 0.27a
Soil Salinity	***	***	***	**	***	**	***
Irrigation Method	***	***	***	**	***	**	***
Soil Salinity ×Irrigation Method	***	***	***	*	***	***	***

* indicates P<0.05 significant level; ** indicates the extremely significant level of P<0.01, the same below. Data are means ± SD (n = 3).

**Table 4:** The 40~60 cm soil layer.

Treatment	HCO_3-_ ( mg·kg^-1^)	Cl^-^ ( mg·kg^-1^)	SO_4_^2-^ (mg·kg^-1^)	Ca^2+^ (mg·kg^-1^)	Mg^2+^ (mg·kg^-1^)	K^+^ (mg·kg^-1^)	Na^+^ (mg·kg^-1^)
MNBs-S_0_	0.40 ± 0.02e	1.22 ± 0.08d	2.03 ± 0.17e	0.38 ± 0.02g	0.23 ± 0.02e	0.14 ± 0.01d	1.34 ± 0.10e
CF-S_0_	0.43 ± 0.02d	0.92 ± 0.05e	1.61 ± 0.14f	0.34 ± 0.01h	0.17 ± 0.01f	0.12 ± 0.01e	0.82 ± 0.07g
MNBs-S_0.3_	0.43 ± 0.02d	1.67 ± 0.13c	3.02 ± 0.23c	0.62 ± 0.04e	0.29 ± 0.03b	0.15 ± 0.01c	2.41 ± 0.21d
CF-S_0.3_	0.44 ± 0.02d	1.24 ± 0.09d	2.14 ± 0.18e	0.58 ± 0.03f	0.24 ± 0.02d	0.13 ± 0.01e	1.03 ± 0.08f
MNBs-S_0.6_	0.46 ± 0.03c	2.31 ± 0.21b	4.33 ± 0.33b	0.71 ± 0.06d	0.31 ± 0.03b	0.17 ± 0.01b	3.62 ± 0.27c
CF-S_0.6_	0.48 ± 0.04b	1.51 ± 0.11c	2.89 ± 0.20d	0.73 ± 0.06c	0.27 ± 0.02c	0.16 ± 0.01c	2.44 ± 0.23d
MNBs-S_0.9_	0.49 ± 0.04b	3.3 ± 0.27a	5.03 ± 0.41a	0.85 ± 0.07a	0.35 ± 0.03a	0.21 ± 0.02a	4.59 ± 0.34a
CF-S_0.9_	0.53 ± 0.05a	2.41 ± 0.23b	3.22 ± 0.24c	0.8 ± 0.07b	0.29 ± 0.03b	0.18 ± 0.01b	3.71 ± 0.28b
Soil Salinity	***	***	***	**	***	**	***
Irrigation Method	***	***	***	**	***	**	***
Soil Salinity ×Irrigation Method	***	***	***	*	***	***	***

* indicates P<0.05 significant level; ** indicates the extremely significant level of P<0.01, the same below. Data are means ± SD (n = 3).

As the salt concentration increased, distinct trends in ion accumulation were observed across soil layers. In the topsoil (0~20 cm), under the same salinity conditions, Micro-Nanobubble Oxygenation Irrigation significantly reduced the concentrations of most ions, with a more pronounced reduction observed at higher salt concentrations. In contrast, non-aerated irrigation resulted in a consistent increase in all ion concentrations with increasing salinity. In both the 20~40 cm and 40~60 cm layers, Micro-Nanobubble Oxygenation Irrigation markedly enhanced the accumulation of Na^+^, Cl^-^, SO_4_²^-^, and HCO_3_^-^, while the concentrations of Ca²^+^, Mg²^+^, and K^+^ remained largely unaffected.

Overall, Micro-Nanobubble Oxygenation Irrigation effectively suppressed the accumulation of most ions in shallow soil, particularly under high-salinity conditions, demonstrating its regulatory capacity on ion dynamics under salt stress. In deeper soil layers, this treatment significantly promoted the accumulation of Ca²^+^, Mg²^+^, K^+^, Na^+^, HCO_3_^-^, Cl^-^, and SO_4_²^-^. The pronounced enhancement effect of Micro-Nanobubble Oxygenation Irrigation highlights its important role in regulating soil ion distribution under high-salinity conditions.

### 3.4 Soil enzyme activity

**Figure 4:**
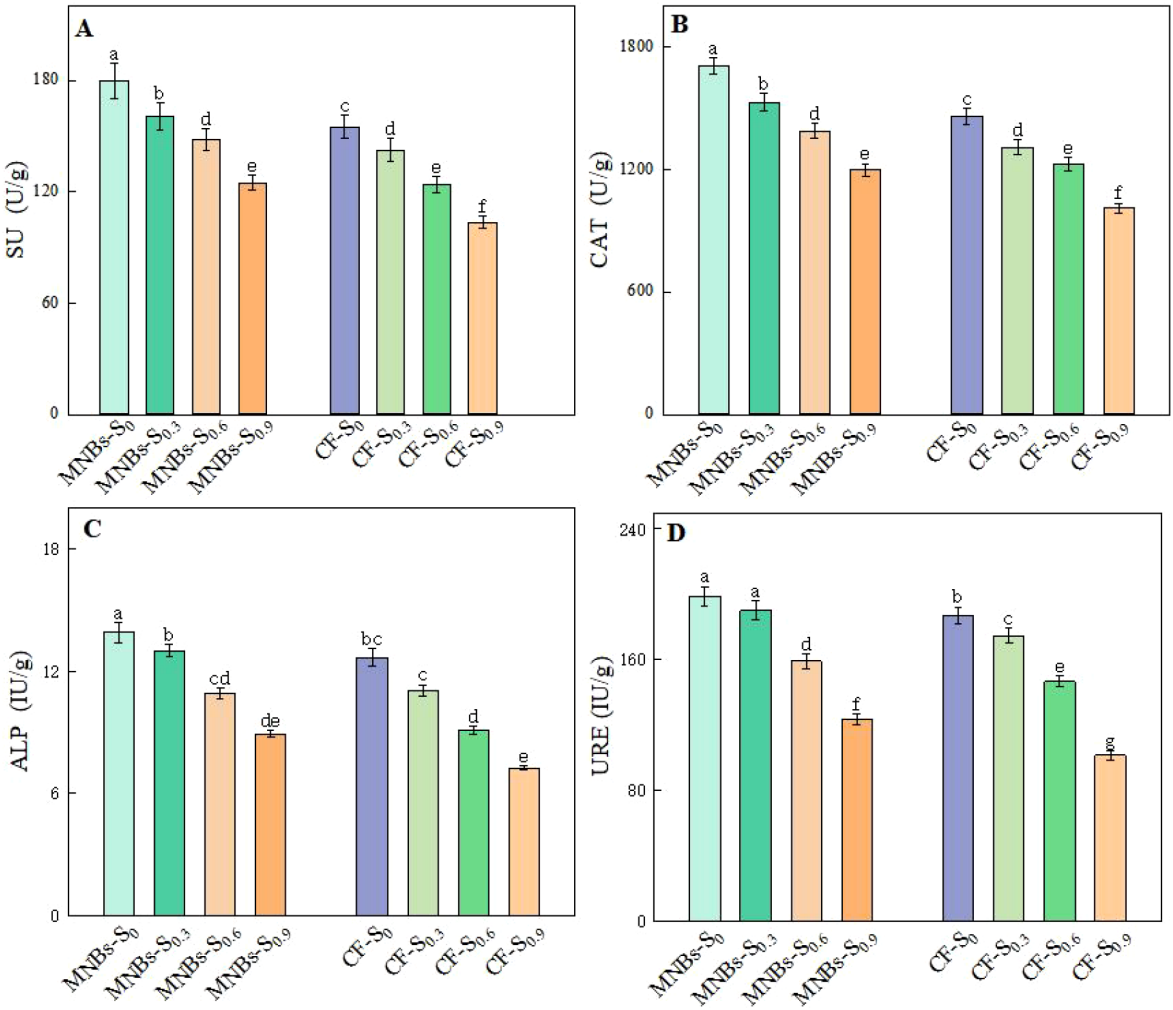
Effects of different treatments on cotton soil enzyme activities. **(A)** Soil sucrase; **(B)** Catalase; **(C)** Alkaline phosphatase; **(D)** urease (URE). Different lowercase letters (a, b, c,…) indicate significant differences in the mean soil enzyme activity levels among treatments (*P* < 0.05), while the same lowercase letters indicate no significant differences in the mean soil enzyme activity levels among treatments (*P* < 0.05).

To investigate the effects of different irrigation treatments on soil enzyme activities in cotton fields (**Figure 4**), the activities of four key enzymes—sucrase (SU), catalase (CAT), alkaline phosphatase (ALP), and urease (URE)—were analyzed under eight treatment combinations in a saline soil environment. The results demonstrated that Micro-Nanobubble Oxygenation Irrigation significantly enhanced the activities of all these enzymes compared to conventional non-oxygenated irrigation.

Specifically, compared to the non-oxygenated control, the MNBs-S_0_ treatment increased SU, CAT, ALP, and URE activities by 6.47%, 3.11%, 5.45%, and 0.30%, respectively (*P* < 0.05). The MNBs-S_0.3_ treatment led to increases of 2.53%, 0.23%, 21.77%, and 0.19% in the same enzymes (*P* < 0.05). Similarly, the MNBs-S_0.6_ treatment elevated enzyme activities by 4.65% (SU), 0.39% (CAT), 0.22% (ALP), and 1.87% (URE) (*P* < 0.05), while the MNBs-S_0.9_ treatment resulted in increases of 7.84%, 0.13%, 7.72%, and 0.58%, respectively (*P* < 0.05).

These findings indicate that Micro-Nanobubble Oxygenation Irrigation can effectively improve the physicochemical properties and biological activity of saline-alkali cotton soils. By enhancing soil enzyme activities, this irrigation technology contributes to improved soil fertility, increased buffering capacity, and the creation of a more favorable soil environment for cotton growth.

### 3.5 Soil microorganisms

#### 3.5.1 Microbial community diversity

**Figure 5:**
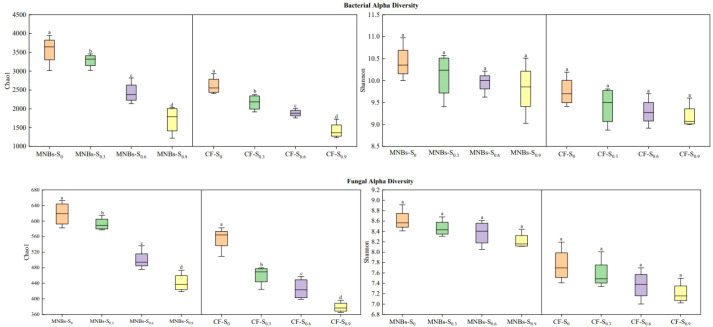
Effects of different treatments on soil microbial community diversity. Bacterial α-diversity was characterized using the Chao1 index **(A)** and Shannon index **(B)**; fungal α-diversity was assessed via the Chao1 index **(C)** and Shannon index **(D)**. Boxes represent interquartile ranges (IQR), with horizontal lines within boxes denoting medians extending to 1.5 times the IQR. Different lowercase letters **(a–c)** indicate significant differences in mean soil enzyme activity levels among treatments (*P* < 0.05). Identical lowercase letters denote no significant differences in mean soil enzyme activity levels among treatments (*P* < 0.05).

This study evaluated the impacts of eight different treatments on bacterial and fungal diversity in cotton field soils(**Figure 5**). The results revealed that elevated soil salinity significantly reduced the bacterial Chao1 index. Under conditions of equivalent soil salinity, Micro-Nanobubble Oxygenation Irrigation markedly increased the bacterial Chao1 index compared to non-oxygenated treatments (*P* < 0.05). In contrast, no significant differences were observed in the bacterial Shannon index across the various treatments.

#### 3.5.2 Microbial community structure

**Figure 6:**
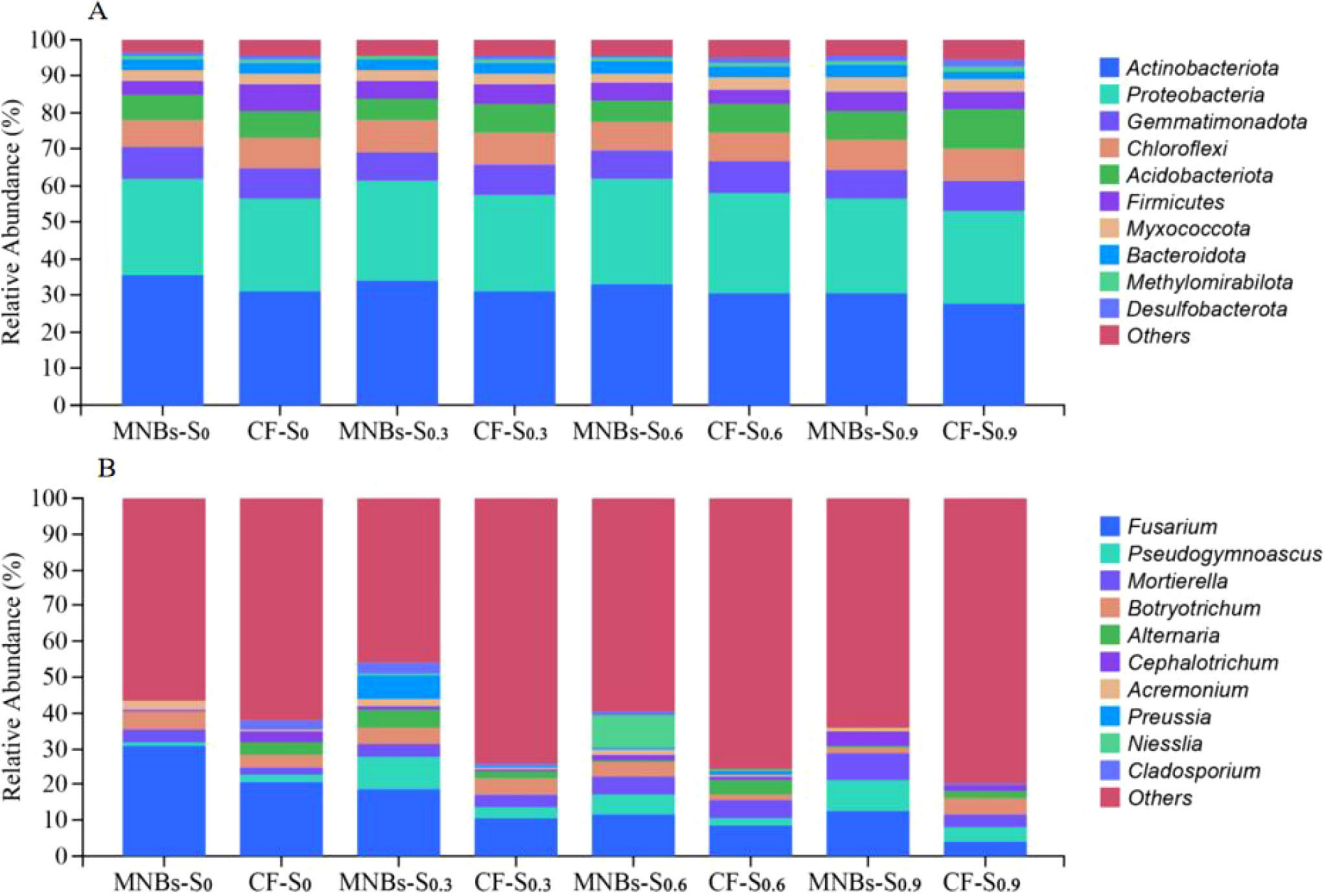
Effects of different treatments on soil microbial community structure. **(A)** Genus-level soil fungal community structure (TOP10); **(B)** Genus-level soil bacterial community structure (TOP10).

This study analyzed soil samples subjected to eight different treatments, in which the top 10 most abundant species were selected for presentation, while the remaining species were grouped as “Others” (**Figure 6**). Among bacteria, the dominant phyla with relative abundances exceeding 5% were, in descending order: Actinobacteriota, Proteobacteria, Gemmatimonadota, Chloroflexi, and Firmicutes. Overall, differences in the relative abundances of dominant bacterial phyla among treatments were relatively small. As shown in the figure, microbial abundance generally decreased with increasing soil salinity. However, Micro-Nanobubble Oxygenation Irrigation increased the abundances of Actinobacteriota and Proteobacteria.

For fungi, the dominant genera with relative abundances exceeding 5% were Fusarium and Pseudogymnoascus. Under conventional irrigation, the abundances of Fusarium and Pseudogymnoascus decreased as soil salinity increased. In contrast, Micro-Nanobubble Oxygenation Irrigation significantly enhanced the abundances of both fungal genera.

### 3.6 Enzyme activity in cotton leaves

**Figure 7:**
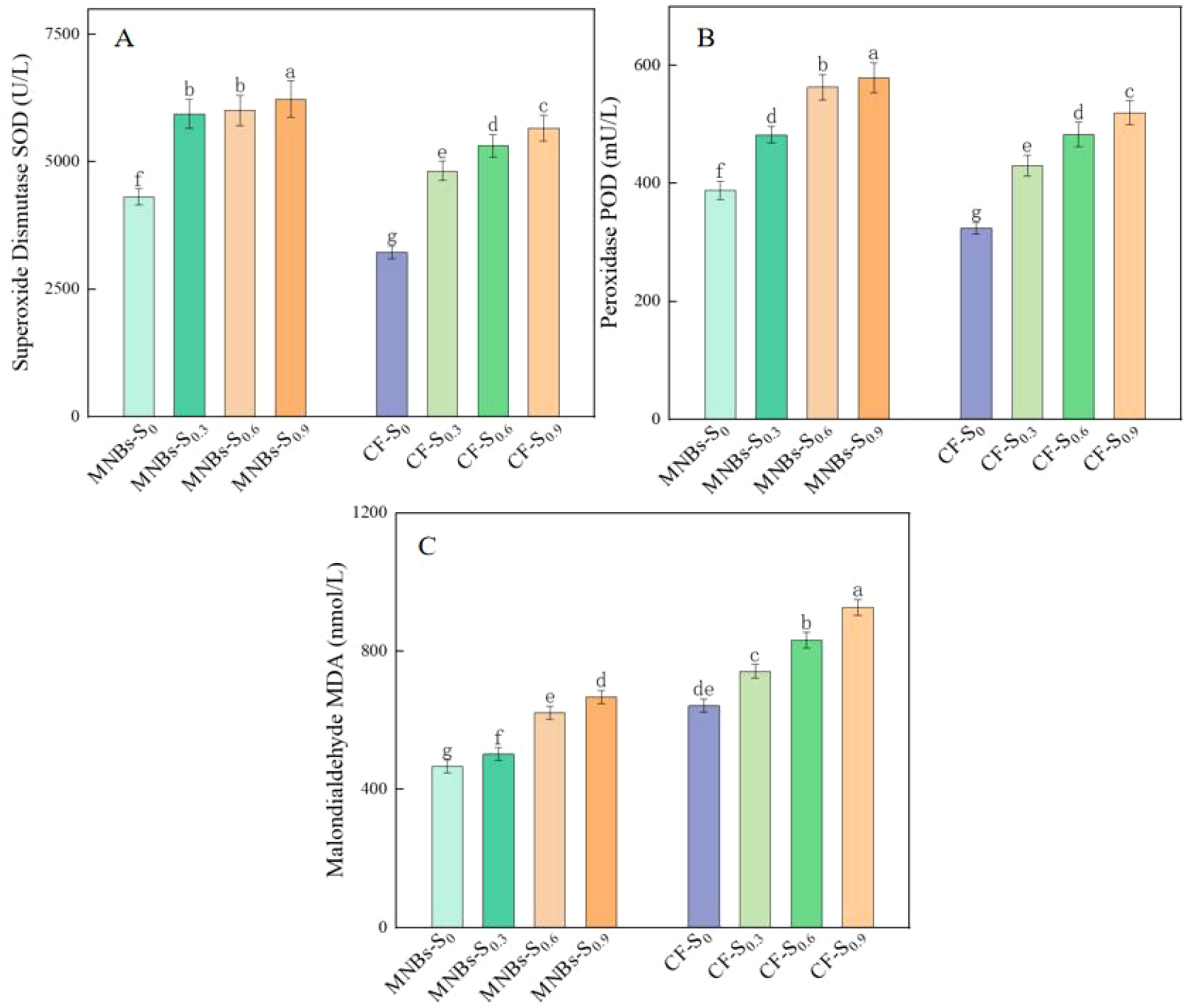
Effects of different treatments on enzyme activities in cotton leaves. **(A)** Superoxide Dismutase (SOD) activity; **(B)** Peroxidase (POD) activity; **(C)** Malondialdehyde (MDA) content. Different lowercase letters (a–c) indicate significant differences in the mean values of cotton growth indices among treatments (*P* < 0.05). The same lowercase letters indicate no significant differences in the mean values of growth indices among treatments (*P* < 0.05).

To investigate the effects of different treatments on enzyme activities in cotton leaves under salt stress (**Figure 7**), this study analyzed superoxide dismutase (SOD) and peroxidase (POD) activities, as well as malondialdehyde (MDA) content, under eight different treatment conditions. As soil salinity increased, both SOD and POD activities in cotton leaves exhibited a gradual upward trend. Micro-Nanobubble Oxygenation Irrigation significantly enhanced SOD and POD activities, which increased by 33.63%, 23.27%, 13.08%, and 10.11% (*P* < 0.05), and by 19.78%, 12.16%, 8.32%, and 19.89% (*P* < 0.05), respectively, compared to the non-oxygenated treatment. In contrast, Micro-Nanobubble Oxygenation Irrigation significantly reduced MDA content, with decreases of 27.38%, 32.24%, 25.23%, and 28.02% (*P* < 0.05) relative to the non-oxygenated control. These results indicate that Micro-Nanobubble Oxygenation Irrigation effectively enhances the antioxidant capacity of cotton under saline conditions.

### 3.7 Cotton growth parameters

**Figure 8:**
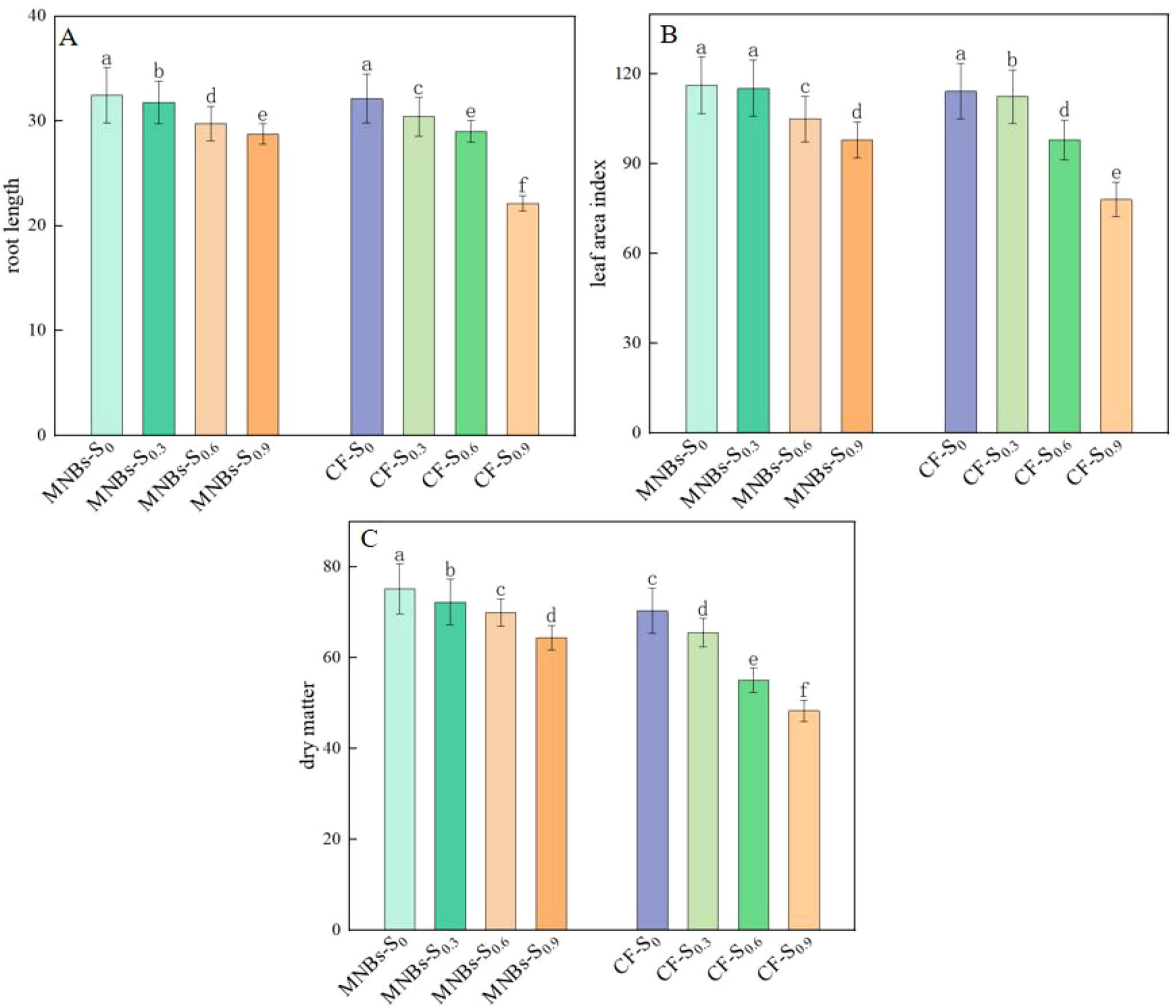
Effect of different treatments on enzyme activity in cotton leaves. **(A)** Cotton root length; **(B)** Leaf area index; **(C)** Dry matter accumulation. Different lowercase letters (a–c) indicate significant differences in the mean values of cotton growth indicators among treatments (*P* < 0.05). The same lowercase letters indicate no significant differences in the mean values of growth indicators among treatments (*P* < 0.05).

To investigate the effects of different treatments on cotton growth parameters under salt stress(**Figure 8**), this study analyzed eight treatment regimens regarding root length, leaf area index, and dry matter accumulation in cotton. As indicated in the results, all growth metrics exhibited a declining trend with increasing soil salinity. However, Micro-Nanobubble Oxygenation Irrigation significantly improved root length, leaf area index, and dry matter accumulation. Specifically, compared to non-oxygenate dirrigation, the Micro-Nanobubble oxygenated treatments (MNBs-S_0_, MNBs-S_0.3_, MNBs-S_0.6_, and MNBs-S_0.9_) increased root length by 0.96%, 2.62%, 4.47%, and 29.90% (*P* < 0.05), and enhanced dry matter accumulation by 6.82%, 10.34%, 27.10%, and 33.29% (*P* < 0.05), respectively. These findings suggest that Micro-Nanobubble oxygenated improves the soil oxygen environment, facilitates root respiration, and thereby promotes dry matter accumulation in cotton under saline conditions.

### 3.8 Cotton yield

This study analyzed the effects of eight different treatments on cotton yield components under salt stress (**Table 5**). The results demonstrated that Micro-Nanobubble oxygenated treatments (MNBs-S_0_, MNBs-S_0.3_, MNBs-S_0.6_, and MNBs-S_0.9_) significantly increased cotton yield by 33.78%, 33.93%, 57.11%, and 59.06%, respectively, compared to the non- oxygenation irrigation treatment (*P* < 0.05). These findings indicate that Micro-Nanobubble oxygenated alleviates the adverse effects of soil salinity stress on cotton, thereby contributing to enhanced cotton yield.

**Table 5:** The impact of oxygenation irrigation on cotton yield.

Treatment	Number of bolls per plant	Weight per boll g ^-1^	Seed cotton yield/ (kg·hm^-2^)
MNBs-S_0_	9.98 ± 1.34a	6.52 ± 0.26a	6978.65 ± 326.14a
CF-S_0_	9.34 ± 0.98a	5.36 ± 0.20a	5216.49 ± 297.19bc
MNBs-S_0.3_	9.86 ± 1.28a	6.34 ± 0.23a	6726.16 ± 311.49a
CF-S_0.3_	8.67 ± 0.82ab	5.22 ± 0.19ab	5022.14 ± 278.61b
MNBs-S_0.6_	8.26 ± 0.73b	4.89 ± 0.18b	4782.92 ± 251.11c
CF-S_0.6_	8.01 ± 0.67c	4.14 ± 0.16b	3044.26 ± 178.59d
MNBs-S_0.9_	7.87 ± 0.52cd	3.79 ± 0.15bc	1915.37 ± 142.28e
CF-S_0.9_	7.26 ± 0.41d	3.16 ± 0.12c	1204.16 ± 134.50f
Soil Salinity	***	***	***
Irrigation Method	***	***	***
Soil Salinity ×Irrigation Method	***	***	***

** indicates *P* < 0.05 significant level; *** indicates the extremely significant level of (*P* < 0.01), the same below. Data are means ± SD (n = 3).

### 3.9 Correlation analysis

**Figure 9:**
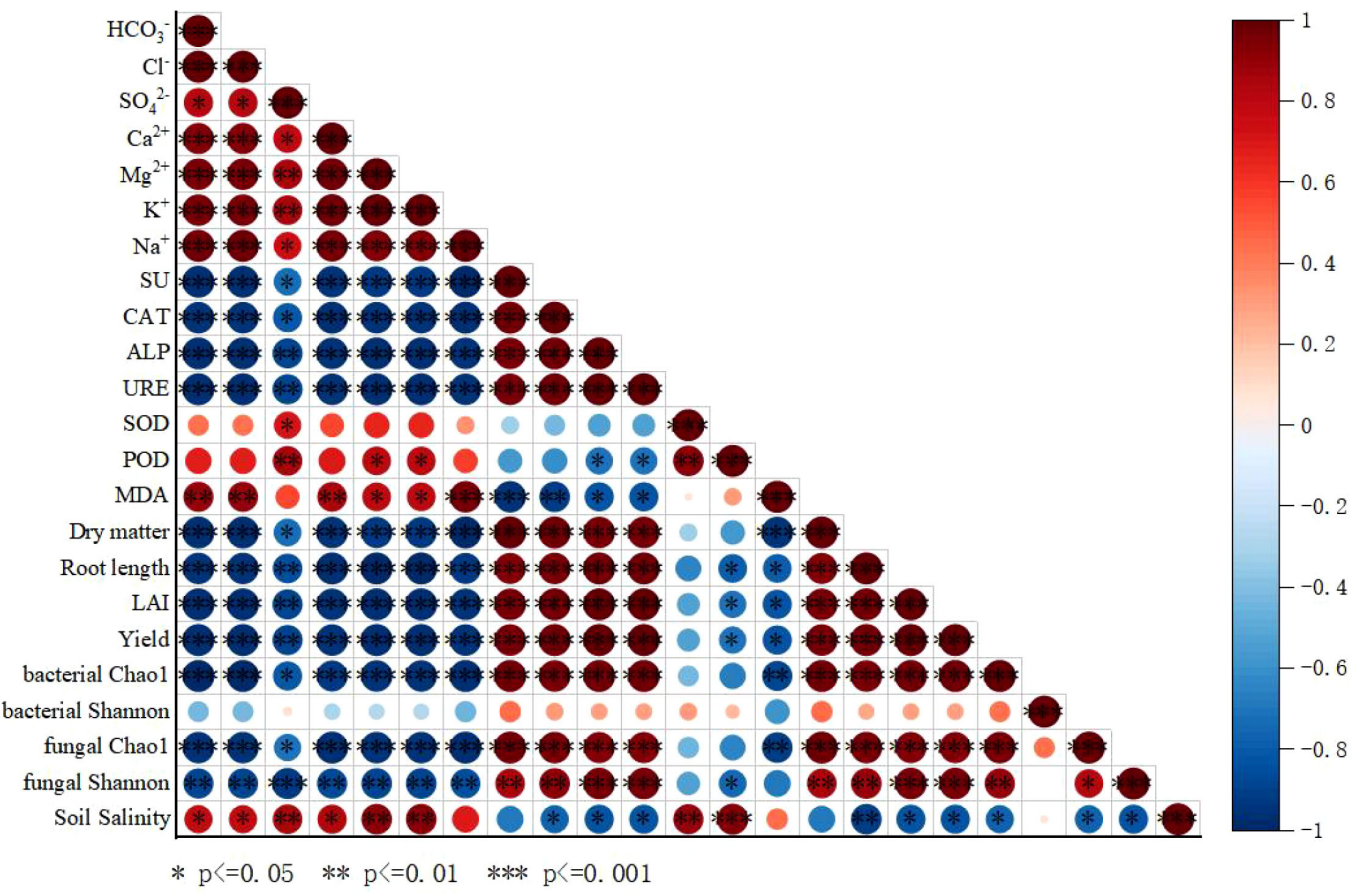
Correlation analysis of treatment indicators: Red bands indicate positive correlations, blue bands indicate negative correlations.

Correlation analysis among soil environmental factors, physiological growth indicators, and yield components in saline cotton fields (**Figure 9**) revealed a highly significant positive correlation (*P* < 0.01) between cotton yield and agronomic traits, indicating that Micro-Nanobubble oxygenated facilitates cotton root elongation, leaf area expansion, and dry matter accumulation. Soil salinity was negatively correlated with the activities of the four soil enzymes, as well as with both bacterial and fungal Chao1 indices, whereas it showed no significant association with bacterial or fungal Shannon indices. Cotton yield was significantly correlated with soil salinity, soil enzyme activities, leaf SOD and POD activities, and bacterial and fungal Chao1 indices. In contrast, no significant correlation was observed between yield and bacterial or fungal Shannon indices, while a negative correlation was identified with malondialdehyde (MDA) content.

## 4 Discussion

Soil salinity stands as one of the primary limiting factors for crop growth and development (Mahmud, 2025).Excessive salt content in soil severely inhibits plant physiological processes and leads to significant yield reductions (Dong et al., 2025). The risk is particularly pronounced in clayey soils. Clay, with its high specific surface area and cation exchange capacity, can adsorb and retain various ions (Farrah et al., 2025). However, as salt concentration increases, the high mobility of Na^+^ in water leads to its rapid accumulation in high-salinity environments, exacerbating soil salt content and posing potential adverse effects on plant growth. Moreover, the small pores in clayey soils tend to trap salts under high-salinity conditions, making plant roots more susceptible to salt stress. To address this challenge, oxygenated irrigation has been shown to effectively enhance soil aeration by promoting soil aggregate formation and improving pore structure (Zhao et al., 2024; Yu et al., 2024; Xu et al., 2024). Notably, oxygenated irrigation increases soil porosity, which can promote the translocation of salts to deeper soil layers, thereby alleviating surface accumulation. Building on this foundation, the present study further reveals that micro-nanobubble oxygenation irrigation significantly reduces salt accumulation in the surface soil layer. Specifically, compared to the non-oxygenated treatment (CF-S_0_), the soil salt content under micro-nanobubble oxygenation irrigation (CF-S_0.3_, CF-S_0.6_, and CF-S_0.9_) decreased by 20.00%, 33.33%, 38.10%, and 41.46%, respectively, demonstrating a remarkable suppression of surface salt accumulation.

These findings align with previous reports by Zheng and Ouyang (Zheng et al., 2025; Ouyang et al., 2025), which indicate that oxygenation improves soil aeration and water retention capacity, thereby enhancing oxygen supply in the root zone. This improvement promotes root respiration and crop growth (Ghosh et al., 2025), enhances salinity tolerance, and mitigates salt-induced damage. Furthermore, micro-nanobubble oxygenation significantly enhances microbial activity in the rhizosphere soil (Xiao et al., 2025). The activated microbial community accelerates organic matter decomposition and releases metabolites that contribute to soil structure optimization, thereby further improving soil permeability and aeration (Pelaez-Sanchez et al., 2024). Collectively, these processes facilitate salt leaching and translocation, ultimately reducing salt content in the surface soil layer.

With increasing soil salinity, the significant accumulation of Na^+^ and Cl^-^ exacerbates secondary salinization. Furthermore, the high cation adsorption capacity of clay minerals markedly reduces the availability of beneficial ions such as Ca²^+^ and Mg²^+^ (Meelua et al., 2025; Batistic and Kudla, 2004). Our results demonstrate that micro-nanobubble oxygenation irrigation effectively alleviated ion imbalance under salt stress. Although the concentrations of Ca²^+^, Mg²^+^, K^+^, Na^+^, HCO_3_^-^, Cl^-^, and SO_4_²^-^ in the topsoil showed an overall increasing trend across salinity levels, their rates of increase were significantly reduced in the oxygenation treatment. Notably, micro-nanobubble oxygenation significantly inhibited the accumulation of Mg²^+^, Na^+^, HCO_3_^-^, and SO_4_²^-^, indicating its regulatory role in soil ion dynamics. This inhibitory effect was particularly pronounced under high-salinity conditions and led to a marked reduction in the concentrations of Na^+^ and Cl^-^. These findings align with those reported by Guo et al (Guo et al., 2025)].The underlying mechanisms involve multiple interrelated physiological and ecological processes. Micro-nanobubble oxygenation enhances root growth and physiological activity, thereby promoting the selective uptake of nutrient ions such as Ca²^+^, Mg²^+^, and K^+^. Simultaneously, it stimulates microbial metabolism, facilitating the secretion of organic acids and extracellular polysaccharides, which can compete with Cl^-^ and SO_4_²^-^ for adsorption sites or form complexes with Na^+^, thus reducing their phytotoxicity. Additionally, by improving soil aggregate stability and pore structure, this technology promotes the leaching of saline ions and reduces their accumulation in the surface layer. Through these synergistic effects, micro-nano bubble oxygenation irrigation effectively regulates ion homeostasis in the root zone, mitigates specific ion toxicity, improves the soil ecological environment, and ultimately enhances the physiological tolerance of crops to salt stress.

Compared to conventional irrigation, micro-nanobubble oxygenation irrigation (MNBs) significantly enhanced the activities of soil enzymes, including sucrase (SU), catalase (CAT), alkaline phosphatase (ALP), and urease (URE). This finding is consistent with the report by Li et al (Li et al., 2013), Previous studies have shown that oxygenation alleviates the inhibition of enzyme activities under anaerobic conditions by improving oxygen distribution in the root zone and stimulating microbial metabolism. Previous studies have shown that MNBs specifically strengthen the functional intensity of key soil enzyme systems, thereby optimizing nutrient transformation and availability, which further confirms the positive impact of oxygenation on the soil biochemical environment (Wang et al., 2024).

In terms of the soil microbial community, the present study revealed that MNBs irrigation under salt stress distinctly reshaped both bacterial and fungal populations. For bacteria, MNBs significantly increased diversity and notably enriched the phyla Actinobacteriota and Proteobacteria. This shift is functionally important: Actinobacteriota enhance soil carbon turnover and can produce phytohormones that stimulate root growth (Shao et al., 2024; Yanes and Bajsa, 2016), while Proteobacteria include key plant-growth-promoting rhizobacteria (PGPR) that facilitate nitrogen fixation and phosphate solubilization, directly improving nutrient availability for plant uptake (Bulgarelli et al., 2013). Consequently, their increased abundance likely underpins the observed improvements in nutrient availability and soil structure, promoting plant health at the root-soil interface.

Regarding fungi, MNBs counteracted the salt-induced decline of dominant genera Fusarium and Pseudogymnoascus. The enrichment of these taxa suggests a shift in rhizosphere dynamics. While Fusarium comprises both pathogenic and saprophytic/endophytic species, and Pseudogymnoascus is primarily saprotrophic (Hirsch, 2018; Wang et al., 2024), their increased abundance under MNBs likely reflects altered carbon cycling and a microbial community adapted to improved oxygenation. Critically, changes in the abundance of such root-associated fungi can influence plant health by modulating organic matter decomposition, competing with pathogens, and interacting with root cells (Shaharoona et al., 2008). Further functional characterization is needed to fully elucidate their role under MNBs irrigation, but their promotion indicates a significant MNBs-induced restructuring of the fungal community with potential implications for plant performance.

Under salt stress, the excessive production of reactive oxygen species (ROS) induces severe oxidative damage in plant cells. Superoxide dismutase (SOD) and peroxidase (POD) serve as the primary enzymatic defense system in cotton leaves, acting synergistically to alleviate ROS-induced oxidative stress (Yadav et al., 2025). SOD catalyzes the conversion of superoxide anions into hydrogen peroxide (H_2_O_2_), while POD further decomposes H_2_O_2_ into water and oxygen, thereby inhibiting the formation of highly reactive hydroxyl radicals. The present study revealed that micro-nanobubble oxygenation irrigation (MNBs) treatments (MNBs-S_0_, MNBs-S_0.3_, MNBs-S_0.6_, and MNBs-S_0.9_) significantly enhanced the activities of SOD and POD, indicating improved antioxidant capacity in crops under saline conditions. This enhancement can be attributed to the ameliorated oxygen supply in the rhizosphere due to micro-nano bubbles. Adequate oxygen promotes mitochondrial respiration and ATP production in roots, supplying essential energy and carbon skeletons for the synthesis of antioxidant enzymes and their cofactors. Moreover, elevated oxygen levels may upregulate the expression of genes encoding SOD and POD via oxygen-sensing signaling pathways, thereby facilitating *de novo* enzyme synthesis (Chen et al., 2025). Such molecular-level responses ensure sustained ROS-scavenging enzymatic activity under prolonged stress. Malondialdehyde (MDA), a terminal product of membrane lipid peroxidation, is widely recognized as a reliable biomarker of oxidative damage (Shaul and Anderson, 1998). In this study, compared to non-aerated controls, MNBs treatments significantly reduced MDA content. Specifically, the MNBs-S_0.9_ treatment resulted in a 28.02% reduction compared to CF-S_0.9_. These results demonstrate that MNBs effectively maintain the structural integrity of cell membranes by alleviating lipid peroxidation. This finding is consistent with Zhu et al (Zhu et al., 2022; Min et al., 2012), who previously reported that oxygenation irrigation reduces MDA accumulation through enhanced ROS detoxification capacity.

Excessive soil salinity not only inhibits crop growth and development but can also lead to significant yield reductions under severe conditions (Zörb et al., 2018). In this study, MNBs treatments outperformed the non-oxygenated control in terms of cotton root length, leaf area, and dry matter accumulation. This finding aligns with the research by Wang et al (Wang et al., 2024b). indicating that MNBs can promote crop growth by effectively reducing soil salt accumulation and optimizing the root zone environment. Notably, compared to the non-oxygenated treatments, MNBs treatments increased seed cotton yield by 33.78%, 33.93%, 57.11%, and 59.06%, respectively. This result not only confirms the promoting effect on biomass accumulation but also indicates that MNBs directly optimized yield components (such as boll number and single boll weight). These findings are consistent with the results of Zhang et al (Zhang et al., 2023). whose study demonstrated that aerated irrigation can enhance water and nutrient use efficiency in saline-alkali soils, thereby improving crop productivity.

Broader Biogeochemical Implications and Considerations for Scalability. While this study demonstrates the short-term efficacy of MNBs in alleviating salt stress and improving crop productivity, its long-term biogeochemical implications warrant careful consideration. Saline soils are often characterized by the accumulation of recalcitrant organic matter and altered nutrient stoichiometry. Our observed enrichment of Actinobacteriota—specialists in decomposing complex organics—suggests that MNBs could accelerate the turnover of this persistent carbon pool. Future research must determine whether this leads to a net loss of soil organic carbon or promotes the formation of more stable humic substances, thereby evaluating MNBs' ultimate impact on long-term soil carbon sequestration.

Furthermore, the stimulation of nitrogen-cycling microorganisms (e.g., within Proteobacteria) indicates enhanced nutrient cycling. While this may improve short-term nitrogen availability, it raises important questions about long-term nutrient use efficiency and potential environmental trade-offs. Specifically, the increased nitrification activity could elevate the risk of nitrate leaching, especially under intensive irrigation, posing a secondary contamination threat to groundwater. Therefore, integrating MNBs with precision nutrient management is crucial to harness its benefits while mitigating such risks.

The scalability of MNBs technology for large-scale agricultural systems must also be evaluated within key biogeochemical constraints. Its efficacy is likely highly dependent on soil texture. Fine-textured clay soils, with smaller pore throats, may better retain micro-nanobubbles, prolonging their oxidative and physicochemical effects. In contrast, coarse sandy soils might experience rapid bubble escape, diminishing treatment longevity and effectiveness. This texture-dependent retention directly influences the technology's biogeochemical footprint, including the duration of oxygen release and the pattern of salt and nutrient translocation.

Finally, region-scale application requires consideration of interactions with local hydrology. Changes in soil solution chemistry and leaching patterns induced by MNBs could influence shallow groundwater quality over time. A comprehensive assessment integrating soil mapping, hydrological modeling, and life-cycle analysis is essential to develop site-specific implementation strategies that maximize agronomic and environmental sustainability. In conclusion, MNBs represent a promising precision tool for saline soil reclamation, but its transition from field-scale success to widespread adoption must be guided by a deeper understanding of these long-term biogeochemical processes and system-level constraints.

## 5 Conclusion

This study confirms that Micro-Nanobubble Oxygenation Irrigation improves the soil environment of saline-affected cotton fields, promoting cotton physiological growth and yield. It enhances the soil environment by effectively reducing soil salinity, activating soil enzyme activity, optimizing the rhizosphere microbial community structure, and promoting cotton root growth and nutrient absorption. This ultimately enhances cotton physiological growth and increases cotton yield. As an innovative irrigation technology, Micro-Nanobubble Oxygenation Irrigation demonstrates significant application potential. It addresses crop yield reduction caused by poor soil aeration in saline-affected soils of arid regions, warranting further promotion in future agricultural management and irrigation optimization.

## Data Availability

The raw data supporting the conclusions of this article will be made available by the authors, without undue reservation.
